# Clinical, pharmacokinetic and pharmacodynamic data for the MEK1/2 inhibitor pimasertib in patients with advanced hematologic malignancies

**DOI:** 10.1038/bcj.2015.103

**Published:** 2015-12-11

**Authors:** F Ravandi, A Pigneux, D J DeAngelo, E Raffoux, J Delaunay, X Thomas, T Kadia, H Kantarjian, J Scheuenpflug, C Zhao, W Guo, B D Smith

**Affiliations:** 1 Department of Leukemia, Division of Cancer Medicine, The University of Texas MD Anderson Cancer Center, Houston, TX, USA; 2 Service des Maladies du Sang, Centre François Magendie, Hôpital du Haut-Lévèque, Pessac, France; 3 Department of Medical Oncology, Dana-Farber Cancer Institute, Boston, MA, USA; 4 Department of Adult Hematology, Hôpital Saint Louis, Paris, France; 5 Department of Hematology, Hôpital Hotel Dieu, Nantes, France; 6 Department of Hematology, Hôpital Edouard Herriot, Lyon, France; 7 Clinical Biomarkers, Merck KGaA, Darmstadt, Germany; 8 Clinical Oncology, Early Development, EMD Serono, Billerica, MA, USA; 9 Global Biostatistics, Hematologic Cancer and BMT, Sidney Kimmel Comprehensive Cancer Center at John Hopkins, Baltimore, MD, USA

The RAS/RAF/MEK/ERK (MAPK) signaling cascade plays a crucial regulatory role in normal cell functions such as proliferation, survival, differentiation, motility and angiogenesis.^1^ This pathway is among the most frequently activated in cancer.^2^ Sustained MAPK activation is important in a number of hematologic malignancies: in acute myeloid leukemia (AML), constitutive MAPK activation due to hyperexpression of ERK, activation of MEK, or downregulation of the ERK phosphatase, PAC1, is common^3^ and markedly elevated pERK levels have been identified in >80% of patients.^4^ Furthermore, *K-Ras* mutations that deregulate MEK/ERK/MAPK signaling appear to be integral to the growth of myeloproliferative neoplasms such as chronic and juvenile myelomonocytic leukemias, which can develop into AML. *Ras* mutations are common in secondary AML derived from myelodysplastic syndrome (MDS) or chronic myelomonocytic leukemia.^5^ Therefore, agents that target the activated MAPK pathway are of therapeutic interest in these malignancies.^6^

Preclinical investigations have confirmed that MEK inhibitors abrogate the myeloproliferative process and restore normal hematopoiesis in *Ras*-mutated myeloproliferative models.^6^ The MEK inhibitor, trametinib, is approved by the Food and Drug Administration for the treatment of V600E-mutated metastatic melanoma, and clinical trials of other MEK inhibitors for multiple myeloma (MM) and various solid tumor types are ongoing.^1^ Pimasertib (MSC1936369B; Merck KGaA, Darmstadt, Germany) is an orally bioavailable small-molecule inhibitor that binds selectively to and inhibits the activity of MEK1/2, preventing the activation of MEK1/2-dependent effector proteins and transcription factors.^7,8^ It has demonstrated promising antitumor activity in preclinical studies,^7, 8^ inhibiting the growth and survival of MM cells *in vitro*, with cytotoxic activity against the majority of MM cell lines regardless of *Ras* and *B-Raf* mutational status. Pimasertib also significantly reduced tumor growth in mice bearing H929 MM xenografts compared with vehicle-treated mice, an effect that correlated with downregulation of pERK1/2.^7^

We report a trial of pimasertib (NCT00957580/EudraCT 2009-010866-49) that was designed to include an initial safety run-in part in patients with advanced hematologic malignancies to establish the maximum tolerated dose (MTD), and a subsequent open-label phase 2 part in older patients with newly diagnosed, poor prognosis AML who were not candidates for intensive chemotherapy. The phase 2 part was not undertaken, in part because limited antileukemic effects observed were in the safety run-in and the estimated probability of observing clinical benefit in phase 2 was low, and we therefore describe the safety run-in part of the trial.

Patients enrolled to the trial were aged⩾18 years and had various types of hematologic malignancy (primary or secondary AML, MDS, relapsed or refractory MM, advanced myeloproliferative disorders, and relapsed, refractory acute lymphocytic leukemia (ALL)) pathologically confirmed according to World Health Organization classification.^9^ Patients had to have had a second or subsequent relapse after standard therapy with no further established treatment options available, be refractory to available therapies, or be newly diagnosed older patients (⩾75 years of age) who were not candidates for intensive chemotherapy. See Supplementary Information for further details.

Patients received pimasertib orally twice daily (BID) according to two discontinuous (days 1–5, 8–12, 15–19 and 22–26 of a 28-day cycle in regimen 1; days 1–21 of a 28-day cycle in regimen 2) and one continuous dosing regimens. Dose escalation followed a classical 3+3 design, with doses of 8–75, 8–90, and 60–75 mg for regimens 1, 2 and 3, respectively. The proposed sample size was ~33 patients per regimen (that is, a maximum of six patients at each dose level, plus three additional patients at the MTD, assuming no need for replacement of patients in the MTD analysis set; Supplementary Figure S1).

The primary objective was to determine the MTD for each dosing regimen, defined as one dose below the level in which a treatment-related dose-limiting toxicity (DLT) occurred in more than one of six patients. Other objectives included assessment of safety (with ocular adverse events of particular interest), pharmacokinetic (PK) profile, preliminary antileukemic activity of pimasertib, changes in pharmacodynamic markers, including pERK in peripheral blood lymphocytes and/or leukemic blasts, and cytogenetics and molecular markers that may be predictive of response to pimasertib or differences in PK profile. Antileukemic activity was evaluated according to International Working Group Response Criteria. Further details regarding study methodology are shown in the Supplementary Information.

In total, 81 patients were enrolled and 80 were treated, 33 in regimen 1, 32 in regimen 2 and 15 in regimen 3. One patient assigned to regimen 2 was undergoing concurrent treatment with hydroxyurea and was excluded (Supplementary Figure S1). Median age was similar across the regimens (64.0, 64.0, and 61.0 years in regimens 1, 2 and 3, respectively) and most patients (82.5%) had AML (*n*=67), of whom 30 had unfavorable cytogenetic results (Supplementary Table S1).

Compliance with pimasertib therapy was good (median⩾95%), and the median duration of exposure was 3.7, 4.6 and 4.0 weeks for patients treated with regimens 1, 2 and 3, respectively (Supplementary Table S2). Pimasertib was also well tolerated. Although all patients treated experienced at least one treatment-emergent adverse event (TEAE; Table 1), grade⩾3 pimasertib-related TEAEs were reported in only 19 patients. DLTs occurred in one patient treated with pimasertib 42 mg BID (Regimen 1), two out of three patients treated with pimasertib 75 mg BID (Regimen 3) and three out of six patients treated with pimasertib 60 mg BID enrolled during the Regimen 3 cohort expansion phase. See Supplementary Tables S3 and S4 for further information on TEAEs. The MTD was not established in regimens 1 and 2, because DLTs could not be assessed owing to disease progression or disease complications at the highest pimasertib dose levels (75 and 90 mg BID for regimens 1 and 2, respectively), and was pimasertib 60 mg BID for regimen 3.

In terms of clinical activity, 39 of 58 evaluable patients had a best overall response of stable disease (SD), with durations up to 64.9 weeks (Table 2). One patient with *N-Ras*-mutant ALL treated with pimasertib 60 mg BID continuously achieved morphological complete remission with incomplete blood count recovery (CRi) for 4.3 weeks and one patient with MDS in the 30 mg BID dose cohort of regimen 1 achieved partial response (PR) as the best overall response. Nine of 15 patients with AML who received pimasertib 60 mg BID according to any regimen achieved SD as their best overall response (Table 2). Of 10 patients who had a ⩾50% reduction of blasts in bone marrow, seven achieved SD (including one each with *JAK2*, *K-Ras* and *FLT3* mutations) and one had a PR, in addition to the patient with a CRi (Supplementary Table S5).

PK analysis showed that pimasertib was rapidly absorbed following single dosing, exhibited dose proportionality within the dose range of 24–75 mg BID, had linear PK over the dose range tested, and did not exhibit a time-dependent effect (Supplementary Figure S2). The half-life at the MTD in regimen 3 (60 mg BID continuous dosing) was ~3 h following single-dose administration (Supplementary Table S6). These observations support the use of a BID dosing regimen of pimasertib.

Modulation of pERK was measured as a marker for MEK activity in peripheral blood lymphocytes and blasts (Supplementary Figures S3a–d). A decrease in pERK relative to baseline was observed during pimasertib treatment with all three regimens, indicating inhibition of MEK1/2, but pERK activity recovery was observed during the washout periods with the intermittent dosing schedules (Supplementary Figures S3b–d). With all three regimens, the extent of pERK inhibition was reduced on completion of the treatment cycle, most significantly with regimen 1.

In summary, the MTD of pimasertib administered continuously was reached at a dose of 60 mg BID with the continuous pimasertib dosing regimen. Although the MTD was not achieved with the intermittent regimens, regimens 1 and 2 were discontinued because regimen 3 was expected to be superior based on improved pharmacodynamics (that is, sustained target inhibition over the complete treatment cycle) with a comparable safety profile. This was shown to be the case based on the pERK activity data and the tolerability of pimasertib, which was similar with all three regimens and characterised by reversible, mainly mild or moderate AEs. However, the pERK data should be interpreted with caution because of the low patient numbers evaluated and because our data provide no insight into whether inhibition of the ERK pathway led to reciprocal activation of the PI3K pathway. Single-agent pimasertib exhibited antileukemic activity, with significant numbers of patients, and >50% of those with AML achieving SD. The only patient to achieve CRi was one of the two patients with ALL, who also had *N-Ras*-mutant disease. Although patient numbers are too low to derive any firm conclusions, the possibility that single-agent pimasertib is effective in this patient population, in which prognosis is poorer than in those with wild-type disease,^10^ is intriguing. The lack of complete response to single-agent pimasertib may be due to incomplete inhibition of the MAPK pathway, possibly because of compensatory PI3K/mTOR signaling pathway activation.^11, 12^ Therefore, while this trial of single-agent pimasertib was terminated early, our data suggest that trials combining agents targeting the MAPK and PI3K/mTOR pathways may be warranted, perhaps also focusing on patients with *Ras*-mutant tumors or tumors in which the MAPK/MEK/ERK pathway is activated by other mechanisms.

## Figures and Tables

**Table 1:** Most common pimasertib-related TEAEs (incidence ⩾10% in any regimen; safety analysis set)

*Treatment-related TEAEs*	*Regimen 1 (*N=*33)*		*Regimen 2 (*N=*32)*		*Regimen 3 (*N=*15)*	
*Patients with at least one event*, n *(%)*	25 (75.8)		21 (65.6)		14 (93.3)	
	*Grade 1/2*	*Grade 3/4*	*Grade 1/2*	*Grade 3/4*	*Grade 1/2*	*Grade 3/4*
Diarrhea	10 (30.3)	–	12 (34.4)	1 (3.1)	6 (40.0)	3 (20.0)
Nausea	7 (21.2)	–	2 (6.3)	–	2 (13.3)	1 (6.7)
Vomiting	4 (12.1)	–	1 (3.1)	–	2 (13.3)	1 (6.7)
Retinal detachment	2 (6.1)	–	5 (15.6)	–	1 (6.7)	–
Blurred vision	4 (12.1)	–	2 (6.3)	–	–	–
Peripheral edema	4 (12.1)	–	2 (6.3)	–	3 (20.0)	–
Fatigue	2 (6.1)	–	3 (9.4)	–	3 (20.0)	–
Face edema	1 (3.0)	–	1 (3.1)	–	3 (20.0)	–
Skin rash	3 (9.1)	–	4 (12.5)	–	4 (26.7)	–
Hypocalcemia	–	–	1 (3.1)	–	2 (13.3)	–
Hyperuricemia	–	–	–	–	2 (13.3)	–
AST increased	4 (12.1)	–	1 (3.1)	–	1 (6.7)	1 (6.7)
Blood ALP increased	2 (6.1)	1 (3.0)	1 (3.1)	–	2 (13.3)	–

Abbreviations: ALP, alkaline phosphatase; AST, aspartate transaminase; TEAE, treatment-emergent adverse event.

**Table 2:** Best overall response and blast response (efficacy analysis set)

*Response characteristic*	*Regimen 1*		*Regimen 2*		*Regimen 3*	
	*60mg BID*	*Overall*	*60mg BID*	*Overall*	*60mg BID*	*Overall*
*AML (N)*	3	26	3	29	9	11
Best overall response, *n* (%)^a^						
SD	3 (100.0)	12 (46.2)	2 (66.7)	17 (58.6)	4 (44.4)	5 (45.5)
PD	0 (0.0)	8 (30.8)	1 (33.3)	5 (17.2)	2 (22.2)	2 (18.2)
Not evaluable	0	6	0	7	3	4
Blast response, *n* (%)						
Yes	0 (0.0)	2 (7.7)	0 (0.0)	4 (13.8)	1 (11.1)	1 (9.1)
No	3 (100.0)	24 (92.3)	3 (100.0)	25 (86.2)	8 (88.9)	10 (90.0)
						
*ALL (N)*	0	1	0	0	1	1
Best overall response, *n* (%)^b^						
CRi	0 (0.0)	0 (0.0)	0 (0.0)	0 (0.0)	1 (100.0)	1 (100.0)
SD	0 (0.0)	1 (100.0)	0 (0.0)	0 (0.0)	0 (0.0)	0 (0.0)
Blast response, *n* (%)						
Yes	0 (0.0)	0 (0.0)	0 (0.0)	0 (0.0)	1 (100.0)	1 (100.0)
No	0 (0.0)	1 (100.0)	0 (0.0)	0 (0.0)	0 (0.0)	0 (0.0)
						
*MDS (N)*	0	3	0	0	2	3
Best overall response, *n* (%)^c^						
PR	0 (0.0)	1 (50.0)	0 (0.0)	0 (0.0)	0 (0.0)	0 (0.0)
PD	0 (0.0)	1 (50.0)	0 (0.0)	0 (0.0)	0 (0.0)	0 (0.0)
Not evaluable	0	1	0	0	2	3
Blast response, *n* (%)						
Yes	0 (0.0)	2 (66.7)	0 (0.0)	0 (0.0)	0 (0.0)	0 (0.0)
No	0 (0.0)	1 (33.3)	0 (0.0)	0 (0.0)	2 (100.0)	3 (100.0)
						
*MPD (N)*	0	1	1	2	0	0
Best overall response, *n* (%)^d^						
SD	0 (0.0)	1 (100.0)	1 (100.0)	1 (100.0)	0 (0.0)	0 (0.0)
Not evaluable	0	0	0	1	0	0
Blast response, *n* (%)						
Yes	0 (0.0)	1 (100.0)	0 (0.0)	0 (0.0)	0 (0.0)	0 (0.0)
No	0 (0.0)	0 (0.0)	1 (100.0)	2 (100.0)	0 (0.0)	0 (0.0)
						
*MM (N)*	1	2	0	1	0	0
Best overall response, *n* (%)^e^						
SD	1 (100.0)	1 (50.0)	0 (0.0)	1 (100.0)	0 (0.0)	0 (0.0)
PD	0 (0.0)	1 (50.0)	0 (0.0)	0 (0.0)	0 (0.0)	0 (0.0)
Blast response, *n* (%)						
Yes	0 (0.0)	0 (0.0)	0 (0.0)	0 (0.0)	0 (0.0)	0 (0.0)
No	0 (0.0)	0 (0.0)	0 (0.0)	0 (0.0)	0 (0.0)	0 (0.0)
Not evaluable	1	2	0	1	0	0

Abbreviations: ALL, acute lymphotic leukemia; AML, acute myelocytic leukemia; BID, twice daily; CI, clinical improvement; CR, complete remission (morphological for AML); CRi, morphological complete remission with incomplete blood count recovery; CY, cytogenetic response; HI, hematologic improvement major; MDS, myelodysplastic syndrome; MM, multiple myeloma; MPD, myeloproliferative disorders; NCR, near complete response; NO, no response; PD, progressive disease; PR, partial response; PS, plateau state; SD, stable disease.

a AML: no patient achieved CR, CRi, PR, or CY.

b ALL: No CR, PR, PD, or CY responses observed. All patients evaluable.

c MDS: No CR, HI, or CY responses observed.

d MPD: No CR, CI, PR, or PD responses observed.

e MM: No CR, NCR, PR, NO, or PS responses observed. All patients evaluable.
