# Supplementary material for: Clinical, pharmacokinetic and pharmacodynamic data for the MEK1/2 inhibitor pimasertib in patients with advanced hematologic malignancies
Source: Blood Cancer J. 2015 Dec 11;5(12):e375–. doi: 10.1038/bcj.2015.103 (PMC4735070; doi:10.1038/bcj.2015.103)

# SUPPLEMENTARY INFORMATION

**LETTER TO THE EDITOR**

# Clinical, pharmacokinetic and pharmacodynamic data for the MEK1/2 inhibitor pimasertib in patients with advanced hematologic malignancies

**Eligibility criteria**

## **Inclusion criteria**

Female and male patients aged ≥ 18 years with one of the following conditions:

- Primary or secondary acute myeloid leukaemia (AML), pathologically confirmed according to World Health Organization (WHO) classification, who meet at least one of the following conditions:
  - second or subsequent relapse after standard therapy, with no established treatment options available
  - refractory to available therapies, for example, failure to achieve complete response after two induction chemotherapy treatments
  - newly diagnosed older patients (≥ 75 years of age), not candidates for intensive chemotherapy.
- Myelodysplastic syndrome (MDS), International Prognostic Scoring System (IPSS) Int-2 or high risk, with resistance or intolerance to standard treatment and not suitable for transplantation.
- Relapsed or refractory multiple myeloma (MM), with failure or intolerance to at least two prior therapies including thalidomide, lenalidomide, and bortezomib.
- Advanced myeloproliferative disorders (MPD) with no treatment options available.
- Acute lymphocytic leukaemia (ALL), relapsed, refractory or intolerant to standard treatment and with no effective treatment options available.

All patients read and understood the informed consent form and were willing and able to provide informed consent. They fully understood requirements of the trial and were willing to comply with all trial visits and assessments. All study-related materials were reviewed and approved by an independent ethics committee/institutional review board.

Patients and their partners (males with female partners of childbearing potential or female patients of childbearing potential) had to be willing to avoid pregnancy during the trial and until 1 month after the last trial drug administration. Adequate contraception as approved by the Investigator (two barrier methods or one barrier method with spermicide or intrauterine device) was to be used 2 weeks prior to, during, and 1 month after the trial. The use of hormonal contraceptives was to be avoided due to a possible drug-drug interaction in female patients of childbearing age. For the purposes of this trial, childbearing potential was defined as “all female patients after puberty unless post-menopausal for at least 2 years, surgically sterile or sexually inactive”.

## **Exclusion criteria**

- ECOG performance status ≥ 3.
- Hyperleukocytosis with > 30x10^9^/l leukaemia blasts in peripheral blood.
- Acute promyelocytic leukaemia [t(15;17)].
- Administration of any antineoplastic therapy within at least 2 weeks or 5 half-lives of that therapy of the first pimasertib dose; no concurrent use of hydoxyurea.
- Participation in other clinical trials within at least 2 weeks of the first pimasertib dose.
- Clinical evidence of active central nervous system (CNS) leukaemia.
- Active and uncontrolled infection including but not limited to known infection with human immunodeficiency virus (HIV), active hepatitis B, or hepatitis C. Patients with an infection receiving antibiotic treatment were permitted into the trial if they were afebrile and haemodynamically stable for 48 h prior to trial entry.
- Major surgery within 2 weeks prior to trial entry.
- Liver function tests above the following limits at screening: total bilirubin > 1.5 x upper limit of normal (ULN) unless related to Gilbert’s syndrome or haemolysis; aspartate aminotransferase (AST) and/or alanine aminotransferase (ALT) > 2.5 x ULN, or for patients with liver involvement AST and/or ALT > 5 x ULN; and serum creatinine > 1.5 x ULN and/or creatinine clearance (CrCl) < 30ml/min at screening (calculation according to Cockroft and Gault formula).
- International normalised ratio (INR) > 1.5 x ULN unless on treatment with warfarin.
- For female patients: pregnancy or breast-feeding.
- History of difficulty swallowing, malabsorption or other chronic gastrointestinal disease or conditions that may hamper compliance and/or absorption of the tested product.
- Significant cardiac conduction abnormalities and/or pacemaker.
- Retinal degenerative disease (hereditary retinal degeneration or age-related macular degeneration), history of uveitis or history of retinal vein occlusion, and/or any medically relevant abnormal findings at the initial ophthalmologic examination.
- Solid tumours with suspected active disease at the time of enrollment. Patients with adequately treated early stage squamous cell carcinoma of the skin, basal cell carcinoma of the skin, or cervical intraepithelial neoplasia (CIN) were eligible for this study.
- Signs and symptoms suggestive of transmissible spongiform encephalopathy, or family members who suffer(ed) from such.
- Other significant disease that in the investigator’s opinion would exclude the patient from the trial.
- Legal incapacity or limited legal capacity.

## **Definition of dose-limiting toxicity**

## DLTs were evaluated during treatment cycle 1 and were defined as:

## a treatment delay of > 2 weeks due to treatment-related AEs

## any grade ≥ 3 non-haematologic toxicity, excluding asymptomatic increases in liver function tests reversible within 7 days and grade 3 vomiting or diarrhoea that did not persist for more than 3 days with optimal therapy.

## **Ocular Assessments**

A qualified ophthalmologist performed a complete ophthalmologic assessment of each patient, including slit lamp examination of the cornea and anterior segment, and determination of intraocular pressure in both eyes. Visual acuity was determined as 20/20 equivalents (for patients with impaired vision, only corrected acuity was determined); both eyes were assessed using the Early Treatment in Diabetic Retinopathy Study (ETDRS) Standards. Visual field was charted for both eyes using the Goldmann visual field test. Colour vision was determined for both eyes using colored plates (Farnsworth 15D or Lanthony desaturated 15D, whichever was available). Fundoscopy included determination of the presence of retinal pigmentation, changes in vasculature, appearance of the fundus and the macula, as well as any other changes. Digital photographic pictures of the fundus were mandatory. The detailed fundus examination required dilation. Optical coherence tomography (OCT) was performed at all equipped sites. OCT was performed at screening, end of cycles 1 and 2, and every other cycle. OCT was mandatory at the time of visual disturbance and was to be repeated regularly until resolution. Fundus fluorescein angiography and electroretinogram were performed as needed for patients experiencing visual disturbances.

**Pharmacokinetic assessments**

Single-dose PK were analysed after the first pimasertib dose in cycle 1 and multiple-dose PK were analysed in cycle 1 on days 19–21 for Regimens 1 and 2 and on day 26 for Regimen 3. Blood samples were taken predose and 0.5, 1.0, 1.5, 2.0, 2.5, 4.0, 6.0, and 10.0 h postdose (cycles 1–3). Concentration-time profiles for pimasertib were obtained based on plasma samples. PK parameters were evaluated according to non-compartmental standard methods using the computer program KINETICA™, Version 4.4.1 or upgrade.

## **Pharmacodynamics methodology: analysis of pERK activity in peripheral blood lymphocytes and leukaemia blasts**

For the analysis of pERK activity in peripheral blood lymphocytes and leukaemia blasts, blood was collected during cycles 1 and 2 (days 1, 26, and 29 [pre-dose] for Regimen 1, and days 1, 19–21, and 29 [pre-dose] for Regimens 2 and 3). For phorbol-12 myristate 13-acetate (PMA) stimulation, 0.5ml whole blood was incubated with 1µM PMA for 30 minutes at 37°C. Cells were lysed and fixed by incubating them at 37°C for 10 min with BD FACS^TM^ Lyse and Fix 1x solution (Becton Dickinson, BD Biosciences, San Jose, CA, USA). Thawed samples were treated with a permeabilising agent (90% ice-cold methanol; (0.9ml) and labeled with an Alexafluor488 conjugated monoclonal antibody (mAb) specific for p-ERK (clone E10; Cell Signaling Technology, Beverly, MA, USA) and CD45-PerCP-cy5.5 (clone 2D1, Becton Dickinson, BD Biosciences, San Jose, CA, USA). Antibody staining was performed for 1 h at room temperature in dark conditions. Samples were acquired and analysed using a FACSCanto II flow cytometer with FACSDiva software (BD Biosciences, BD Biosciences, San Jose, CA, USA), which allowed differential analysis of lymphocytes and leukaemia blasts.

Median fluorescence intensity (MFI) of pERK in lymphocytes was used to calculate the percentage of pERK induced by PMA:

PpERKi(t) = 100 x (PMA_(t)_ – unstimulated_(t)_) / unstimulated_(t)_ / unstimulated_(t)._

From this, the percentage of pERK modulation versus baseline was calculated:

PMpERK_(t)_ = 100 x PpERKi_(t)_ / PpERKi_(baseline)._

pERK fold increase (FI) in MFI induced by PMA at timepoint, t (see Figure 3a) was calculated as:

$$pERK^{FI-PBMC}(t)=\frac{MFI_{PMA}-MFI_{Control}}{MFI_{Control}}$$

where MFI_PMA_ = MFI of pERK upon PMA stimulation and MFI_control_ = MFI of pERK under control conditions (DMSO).

Modulation at each time point (see Figures 3b, c, and d) was calculated as:


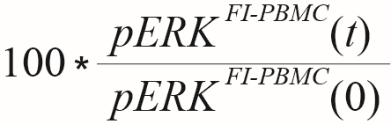


The assay was fully validated before clinical sample analyses (unpublished results).

## **Mutational analyses**

Detection of *K-Ras* mutations was carried out using the Therascreen *K-Ras* PCR Kit (QIAGEN, Manchester Ltd, Manchester, UK, EU), which is based on a panel of 7 real-time PCR assays able to detect the presence of different mutations present in codons 12 and 13 of the *K-Ras* gene. *N-Ras* mutations were assessed using the Therascreen NRAS Pyro Kit using the PyroMark Q24 Instrument (QIAGEN, Manchester Ltd, Manchester, UK), which is able to identify all major mutations of codons 12, 13, and 61. Detection of *JAK2* mutation was carried out using the MutaScreen Ez Kit (Ipsogen, SA, Marseille, France), which is based on a real-time PCR allelic discrimination assay for the detection of the single point mutation G1849T (V617F). Detection of *FLT3* mutations were carried out using the LeukoStrat *FLT3* Mutation Assay (InVivoScribe Technologies, San Diego, CA, USA) to detect the *FLT3* internal tandem duplications (ITDs) and the tyrosine kinase domain (TKD) mutation D835. All the above assays were fully validated before clinical sample analyses.

**Cytogenetic assessment**

Cytogenetic data were reported as follows for patients with AML: 1) favorable: t(8;21)(q22;q22), inv(16)(p13q22), or t(16;16)(p13;q22); 2) intermediate: normal karyotype, –Y, del(9q), +8, +11, or +13; 3) unfavorable: del(5q), –5, –7, del(7q), abn 3q, 9q, 11q, 20q, 21q, 17p, t(6;9), t(9;22) or complex karyotypes (≥ 3 unrelated abnormalities); and 4) unknown: unknown or other cytogenetic abnormalities.^1–3^

**References**

1. Grimwade D, Walker H, Oliver F, Wheatley K, Harrison C, Harrison G*,* *et al.* The importance of diagnostic cytogenetics on outcome in AML: analysis of 1,612 patients entered into the MRC AML 10 trial. The Medical Research Council Adult and Children's Leukaemia Working Parties. *Blood* 1998; **92** :2322–2333.

2. Grimwade D, Walker H, Harrison G, Oliver F, Chatters S, Harrison CJ*, et al.* The predictive value of hierarchical cytogenetic classification in older adults with acute myeloid leukemia (AML): analysis of 1065 patients entered into the United Kingdom Medical Research Council AML11 trial. *Blood* 2001; **98**: 1312–1320.

3. Slovak ML, Kopecky KJ, Cassileth PA, Harrington DH, Theil KS, Mohamed A*, et al.* Karyotypic analysis predicts outcome of preremission and postremission therapy in adult acute myeloid leukemia: a Southwest Oncology Group/Eastern Cooperative Oncology Group Study. *Blood* 2000; **96**: 4075–4083.

Table S1. Baseline characteristics (ITT population)

| *Characteristics* | *Regimen 1* | *Regimen 2* | *Regimen 3* |
| --- | --- | --- | --- |
| *N* | 33 | 33 | 15 |
| *Median age, years (range)* | 64.0 (22–80) | 64.0 (22–78) | 61.0 (33–79) |
| ≥ 75, *n* (%) | 6 (18.2) | 4 (12.1) | 3 (20.0) |
| *Sex, male/female (%)* | 72.7/27.3 | 60.6/39.4 | 53.3/46.7 |
| *ECOG, n (%)* |  |  |  |
| 0 | 4 (12.1) | 9 (27.3) | 4 (26.7) |
| 1 | 22 (66.7) | 18 (54.5) | 5 (33.3) |
| 2 | 7 (21.2) | 6 (18.2) | 6 (40.0) |
| *Hematologic malignancy, n (%)* |  |  |  |
| AML | 26 (78.8) | 30 (90.9) | 11 (73.3) |
| MM | 2 (6.1) | 1 (3.0) | 0 (0.0) |
| MPD | 1 (3.0) | 2 (6.1) | 0 (0.0) |
| MDS | 3 (9.1) | 0 (0.0) | 3 (20.0) |
| ALL | 1 (3.0) | 0 (0.0) | 1 (6.7) |
| *AML cytogenetic results, n (%)^a^* |  |  |  |
| Favourable | 2 (7.7) | 3 (10.7) | 3 (27.3) |
| Unfavourable | 9 (34.6) | 14 (50.0) | 7 (63.6) |
| Intermediate | 14 (53.8) | 11 (39.3) | 1 (9.1) |
| Indeterminate | 1 (3.8) | 0 (0.0) | 0 (0.0) |
| *Prior anti-cancer therapy, n (%)* |  |  |  |
| ≥ 1 type of therapy | 32 (97.0) | 33 (100.0) | 14 (93.3) |
| Systemic therapy | 30 (90.9) | 31 (93.9) | 13 (86.7) |
| Surgery (or other procedure) | 19 (57.6) | 20 (60.6) | 10 (66.7) |
| Bone marrow transplant | 10 (30.3) | 8 (24.2) | 8 (53.3) |
| Radiotherapy | 6 (18.2) | 2 (6.1) | 4 (26.7) |
| *Mutational status, n (%)^b^*  *KRAS*  *NRAS*  *FLT3*  *JAK2* | 16  1 (6.3)  1 (6.3)  2 (12.5)  1 (6.3) | 21  2 (9.5)  1 (4.8)  5 (23.8)  1 (4.8) | 6  0  1 (16.7)  0  0 |
| Abbreviations: ALL, acute lymphotic leukaemia; AML, acute myelocytic leukaemia; ECOG, Eastern Cooperative Oncology Group; ITT. Intent to treat; MDS, myelodysplastic syndrome; MM, multiple myeloma; MPD, myeloproliferative disorders.  ^a^Cytogenetic results for two patients with AML in Regimen 2 are missing. ^b^Percentages are given based on the number of patients with the mutation as a percentage of the total number of patients whose mutational status was assessed. | | | |

Table S2. Exposure to pimasertib – all regimens (safety analysis set)

| *Characteristics* | *Regimen 1 (n = 33)* | *Regimen 2 (n = 32)* | *Regimen 3 (n = 15)* |
| --- | --- | --- | --- |
| *Time on trial (weeks)* |  |  |  |
| Median (range) | 8.6 (2.1–47.9) | 8.4 (2.6–147.6) | 7.4 (5.7–17.6) |
| *Time on treatment (weeks)^a^* |  |  |  |
| Median (range) | 3.7 (0.1–45.1) | 4.6 (0.3–141.0) | 4.0 (1.0–13.1) |
| ≤ 4 weeks | 17 (51.5) | 15 (46.9) | 9 (60.0) |
| > 4 weeks | 16 (48.5) | 17 (53.1) | 6 (40.0) |
| *Total no. of initiated cycles* |  |  |  |
| Median (range) | 1 (1–12) | 2 (1–36) | 1 (1–4) |
| *Cumulative dose (mg)^b^* |  |  |  |
| Median (range) | 1680 (23–28 860) | 1525 (270–46 068) | 3300 (600–10 980) |
| Treatment compliance (%)^c^ |  |  |  |
| Median (range) | 95.6 (50–116) | 97.6 (66–20.7) | 97.5 (52–100) |
| < 90% | 12 (36.4) | 9 (28.1) | 2 (13.3) |
| ≥ 90% | 21 (63.6) | 23 (71.9) | 13 (86.7) |
| ^a^Time on treatment (weeks) = (last dosing date – first dosing date + 1) / 7. ^b^Cumulative dose = sum of all actual (non-zero) daily doses received during the trial. ^c^Treatment compliance = (cumulative dose [mg] / planned cumulative dose [mg]) x 100%. | | | |

**Table S3.** Summary of TEAEs (safety analysis set)

|  | *Regimen 1*  *(n = 33)* | *Regimen 2*  *(n = 32)* | *Regimen 3*  *(n = 15)* |
| --- | --- | --- | --- |
| *N* (%) of patients with |  |  |  |
| Any DLT | 1 (3.0) | 0 (0.0) | 5 (33.3) |
| At least one TEAE | 33 (100.0) | 32 (100.0) | 15 (100.0) |
| At least one TEAE, grade ≥ 3 | 24 (72.7) | 28 (87.5) | 14 (93.3) |
| At least one pimasertib-related   TEAE | 25 (75.8) | 21 (65.6) | 14 (93.3) |
| At least one pimasertib-related   TEAE, grade ≥ 3 | 5 (15.2) | 4 (12.5) | 10 (66.7) |
| At least one ocular TEAE | 9 (27.3) | 13 (40.6) | 7 (46.7) |
| At least one skin/rash TEAE | 16 (48.5) | 13 (40.6) | 9 (60.0) |
| At least one serious TEAE | 24 (72.7) | 28 (87.5) | 12 (80.0) |
| At least one pimasertib-related   serious TEAE | 5 (15.2) | 6 (18.8) | 6 (40.0) |
| A serious TEAE with fatal  outcome | 6 (18.2) | 6 (18.8) | 5 (33.3) |
| At least one pimasertib-related   serious TEAE with fatal outcome | 0 (0.0) | 1 (3.1) | 2 (13.3) |
| At least one TEAE leading to  permanent treatment   discontinuation | 7 (21.2) | 7 (21.9) | 7 (46.7) |
| At least one related TEAE leading   to permanent treatment   discontinuation | 2 (6.1) | 3 (9.4) | 4 (26.7) |
| Abbreviations: DLT, dose-limiting toxicity; TEAE, treatment-emergent adverse event. | | | |

**Table S4.** Most commonly occurring (> 10%) TEAEs (safety analysis set; all grades)

| *TEAEs occurring in ≥ 10% of patients in any regimen (all grades)* | *Regimen 1*  *(N = 33)* | *Regimen 2*  *(N = 32)* | *Regimen 3*  *(N = 15)* |
| --- | --- | --- | --- |
| *Patients with at least one event, n (%)* | 33 (100) | 32 (100) | 15 (100) |
| Diarrhoea | 15 (45.5) | 17 (53.1) | 10 (66.7) |
| Skin rash | 10 (30.3) | 8 (25.0) | 7 (46.7) |
| Asthenia/fatigue | 9 (27.3) | 10 (31.3) | 6 (40.0) |
| Pyrexia/hyperthermia | 8 (24.2) | 11 (34.4) | 2 (13.3) |
| Nausea | 10 (30.3) | 7 (21.9) | 3 (20.0) |
| Oedema peripheral | 6 (18.2) | 8 (25.0) | 5 (33.3) |
| AST increased | 8 (24.2) | 8 (25.0) | 3 (20.0) |
| Febrile neutropenia | 6 (18.2) | 11 (34.4) | 2 (13.3) |
| Pneumonia | 9 (27.3) | 4 (12.5) | 4 (26.7) |
| Visual disturbances | 6 (18.2) | 8 (25.0) | 3 (20.0) |
| SRD | 3 (9.1) | 9 (28.1) | 4 (26.7) |
| Vomiting | 7 (21.2) | 7 (21.9) | 2 (13.3) |
| Dizziness/vertigo/presyncope | 8 (24.2) | 5 (15.6) | 2 (13.3) |
| Sepsis/bacteraemia | 7 (21.2) | 7 (21.9) | 1 (6.7) |
| Hypotension | 6 (18.2) | 7 (21.9) | 1 (6.7) |
| Epistaxis | 7 (21.2) | 5 (15.6) | 1 (6.7) |
| ALT increased | 6 (18.2) | 5 (15.6) | 1 (6.7) |
| Hypoalbuminaemia | 5 (15.2) | 3 (9.4) | 3 (20.0) |
| Hypokalaemia | 4 (12.1) | 5 (15.6) | 2 (13.3) |
| Hyperuricaemia | 3 (9.1) | 4 (12.5) | 3 (20.0) |
| Face/periorbital oedema | 1 (3.0) | 3 (9.4) | 4 (26.7) |
| Hypocalcaemia | 1 (3.0) | 2 (6.3) | 4 (26.7) |
| Pleural effusion | 2 (6.1) | 0 (0.0) | 3 (20.0) |
| Abbreviations: ALT, alanine transaminase; AST, aspartate transaminase; SRD, serous retinal detachment; TEAE, treatment-emergent adverse event. | | | |

**Table S5.** Characteristics of participants with ≥ 50% reduction in blasts in bone marrow and best response to pimasertib treatment

| *Patient* | *Regimen* | *Dose, mg, BID* | *Disease* | *Cytogenetics* | *Mutational status* | *Maximum % reduction from baseline in bone marrow blast* | *Best overall response* | *Time to response (days)* | *Duration of best response (days)* |
| --- | --- | --- | --- | --- | --- | --- | --- | --- | --- |
| 1 | 1 | 23 | MPD | Unknown | *JAK2* | –90.0 | SD | 29 | 46 |
| 2 | 1 | 23 | MDS | Unknown | *K-Ras* | –72.7 | NK |  |  |
| 3 | 1 | 30 | AML | Unfavourable | WT | –58.3 | SD | 29 | 50 |
| 4 | 1 | 30 | MDS | Unknown | WT | –83.3 | PR | 29 | 175 |
| 5 | 1 | 42 | AML | Intermediate | WT | –73.8 | SD | 30 | 1 |
| 6 | 2 | 8 | AML | Unfavourable | WT | –65.9 | SD | 26 | 28 |
| 7 | 2 | 60 | MPD | Unknown | *K-Ras* | –50.0 | SD | 29 | 28 |
| 8 | 2 | 75 | AML | Unfavourable | WT | –71.4 | SD | 29 | 28 |
| 9 | 2 | 75 | AML | Favourable | *FLT3* | –91.2 | SD | 29 | 27 |
| 10 | 3 | 60 | ALL | Unknown | *N-Ras* | –89.7 | CRi | 30 | 30 |
| Abbreviations: ALL, acute lymphotic leukaemia; AML, acute myelocytic leukaemia; BID, twice daily; MDS, myelodysplastic syndrome; MPD, myeloproliferative disorder; NK, not known; PR, partial remission; SD, stable disease; WT, wild-type.  Favourable: t(8;21)(q22;q22), inv(16)(p13q22), or t(16;16)(p13;q22).  Intermediate: normal karyotype, –Y, del(9q), +8, +11, or +13.  Unfavourable: del(5q), –5, –7, del(7q), abn 3q, 9q, 11q, 20q, 21q, 17p, t(6;9), t(9;22), or complex karyotypes (≥ 3 unrelated abnormalities).  Indeterminate: unknown or other cytogenetic abnormalities. | | | | | | | | | |

**Table S6.** Pharmacokinetic parameters for single dose and at steady state following multiple doses of pimasertib at the MTD (60 mg BID,
Regimen 3)

|  | *Statistic* | *C_max_ (ng/ml)* | *T_max_*  *(h)* | *AUC_0-t_*  *(h*ng/ml)* | *AUC0-_∞_*  *(h*ng/ml)* | *CL/f*  *(L/h)* | *V/f*  *(L)* | *T_1/2_*  *(h)* |
| --- | --- | --- | --- | --- | --- | --- | --- | --- |
| *Single dose*  *Cycle 1, Day 1* | GeoMean | 283.8 | 1.102 | 927.3 | 1160.8 | 51.688 | 225.1 | 3.019 |
|  | CV% (n) | 53.8 (12) | 32.6 (12) | 62.4 (12) | 54.1 (11) | 54.1 (11) | 53.9 (11) | 23.9 (11) |
|  | Range | 138–724 | 0.53–2.00 | 319–2,508 | 622–3,040 | 19.74–96.43 | 103–456 | 1.92–3.96 |
| *Multiple dose^a^*  *Cycle 1, Day 21* | GeoMean | 261.83 | 1.75 | 1010.2 | 952.1 | 65.53 | 371.98 | 3.53 |
|  | CV%, (n) | 37.3 (8) | 128.2 (8) | 62.7 (8) | 43.2 (4) | 44.0 (5) | 52.4 (4) | 23.9 (4) |
|  | Range | 135.2–389.8 | 0.5–5.8 | 352–2021 | 544–1462 | 42.5–129.1 | 223.4–730.2 | 2.6–4.5 |
| Abbreviations: AUC_0-t_, area under the curve from time zero to the last sampling time at which the concentration is at or above the lower limit of quantification; AUC0-_∞_, area under the plasma concentration-time curve from time zero extrapolated to infinity; BID, twice daily; C_max_, maximum concentration; CL/f, total body clearance from plasma following oral administration; CV, coefficient of variation (geometric mean); GeoMean, geometric mean; MTD, maximum tolerated dose; t_1/2_, apparent terminal half-life; T_max_, time to maximum concentration; V/f, apparent volume of distribution following oral administration.  ^a^Steady state conditions. | | | | | | | | |

**Figure S1**. Patient disposition and flow through the study


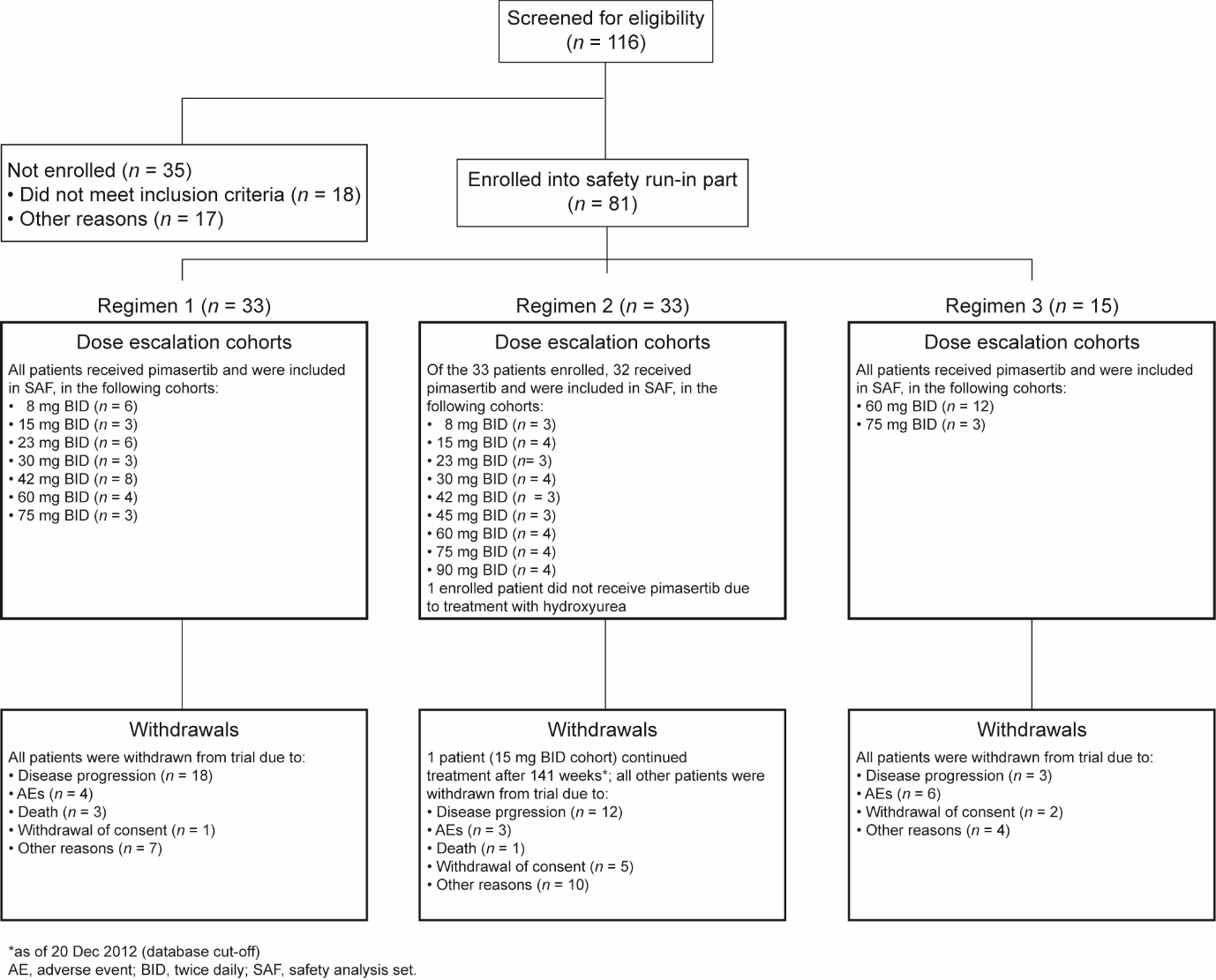


**Figure S2.** Scatter plots of C_max_ versus dose for **a**) Regimen 1, **b**) Regimen 2 and **c**) Regimen 3.


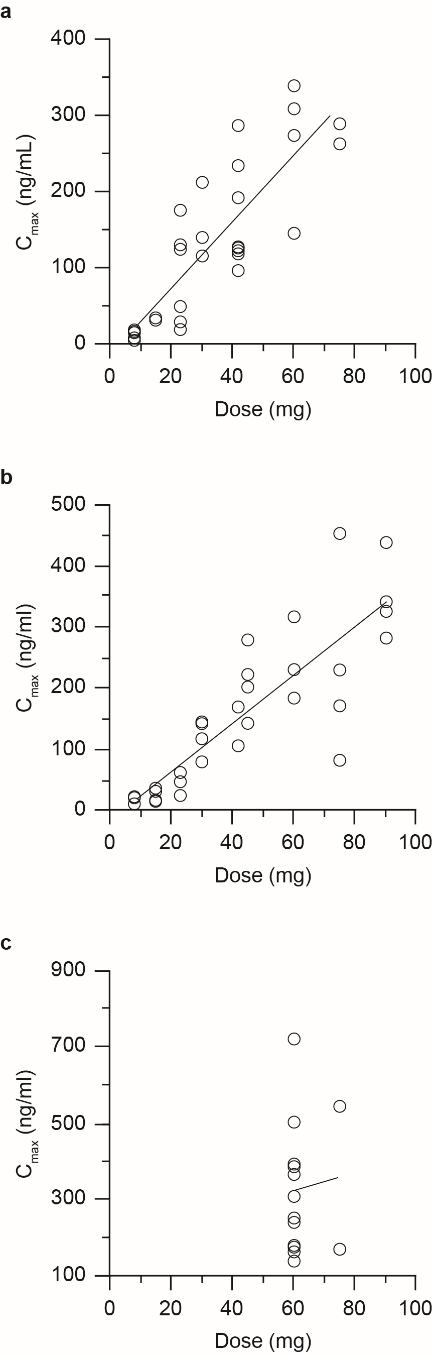


**Figure 3.** Inhibition of pERK in peripheral blood lymphocytes and blasts of patients treated with pimasertib 60 mg BID. (**a**) Day 1 fold increase of *ex-vivo* PMA-stimulated pERK levels prior to pimasertib administration in 19 patients. Predose pERK levels represent baseline values for each patient. (**b**), (**c**) and (**d**): pERK inhibition relative to baseline in Regimens 1, 2 and 3, respectively. Patient numbers are shown above the bars in **b**–**d**. No samples were available on days 19–21 in Regimen 1 (**b**).
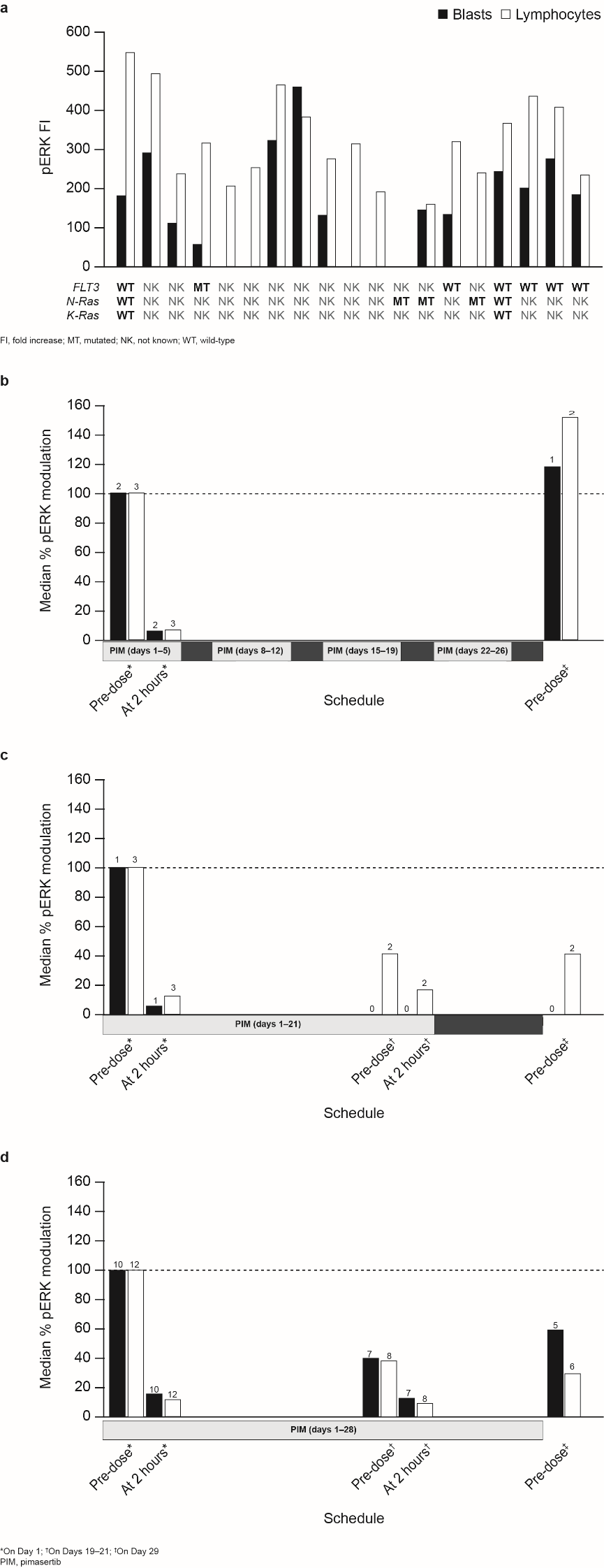

Supplement: Supplementary Information [file bcj2015103x1.docx]
